# Mouse adaptation of influenza B virus increases replication in the upper respiratory tract and results in droplet transmissibility in ferrets

**DOI:** 10.1038/srep15940

**Published:** 2015-11-03

**Authors:** Eun-Ha Kim, Su-Jin Park, Hyeok-Il Kwon, Se Mi Kim, Young-il Kim, Min-Suk Song, Eun-Ji Choi, Philippe Noriel Q. Pascua, Young-Ki Choi

**Affiliations:** 1 Microbiology Department, College of Medicine and Medical Research Institute, Chungbuk National University, 12 Gaeshin-Dong Heungduk-Ku, Cheongju 361-763, Republic of Korea; 2 Department of Infectious Disease, St. Jude Children’s Research Hospital, Memphis, Tennessee 38105-2794

## Abstract

To investigate the molecular changes that allow influenza B viruses to adapt to new mammalian hosts, influenza B/Florida/04/2006 was serially passaged in BALB/c mice until highly virulent. The viral factors underlying this transition were then investigated in mice and ferrets. Five viruses, including the wild-type virus (P0), three intermediate viruses (P5, P9, and P12), and a lethal mouse-adapted virus (P17 (MA)), harbored one to five amino acid substitutions in the hemagglutinin, M, NP, and PA segments suggesting that these mutations enhance virulence. The P17 (MA) virus replicated significantly more efficiently than the P0 virus both *in vitro* and *in vivo* (P < 0.0001), and was highly virulent (MLD_50_: 10^5.25^TCID_50_) while the P0, P5, and P9 viruses did not kill any infected mice (MLD_50_ > 10^6.0^TCID_50_). Furthermore, the P17 (MA) virus grew to greater titers in the ferret upper respiratory tract compared with the P0 and intermediate viruses, and only the P17 (MA) virus was transmissible between ferrets via both direct and aerosol contact. To our knowledge, this is the first study to demonstrate ferret-to-ferret transmission of influenza B virus and to delineate factors that may affect its transmission.

Influenza A and B viruses cause global outbreaks of mild-to-severe respiratory disease each year and are among the most significant public health problems worldwide. The influenza A viruses can be divided into subtypes depending on the antigenicity of their two surface glycoproteins, hemagglutinin (HA) and neuraminidase (NA)^1^. In contrast, the influenza B virus is not divided into subtypes and has only two genetically distinct lineages in humans, B/Vitoria/2/87-like (Victoria lineage) and B/Yamagata/16/88-like (Yamagata lineage). The B/Victoria lineage was predominant during the 1980s, while the B/Yamagata lineage was predominant throughout most of the world during the 1990s^2^. However, a recent study revealed that one of each influenza B linage viruses has been predominantly circulating approximately once every three years with genetic shift and antigenic drift^3^. While influenza B viruses are believed to cause milder clinical disease in mammalian hosts than influenza A viruses, these viruses are essential components of influenza vaccines administered to susceptible groups, such as children and young adults^4^. In most years, the vaccination process is successful; however, antigenic drift and mismatches between circulating viruses and selected vaccine strains have occurred with significant consequences^5^, and little cross-protection between lineages is considered to exist^4^. In this regard, during the 2007–2008 flu seasons, the selected vaccine strains protected against infection with the B/Victoria lineage, but over 95% of the circulating influenza B viruses were of the B/Yamagata lineage^6^. Thus, one proposed alternative to current vaccine strategies is the production of a quadrivalent influenza vaccine (QIV) that includes both influenza B virus lineages^7^.

Previous reports of influenza B virus isolation from dogs, a pig, and harbor seals have not met established criteria for determining whether an infection has occurred, and the virus has shown little-to-no adaptation to these hosts. In fact, influenza B viruses isolated from non-human species have been found to be closely related to concurrently circulating human strains^8^,^9^,^10^. A natural reservoir of influenza B viruses outside humans has not been identified and thus, these viruses are considered to be exclusively human pathogens. Although both influenza A and B viruses have caused widespread human epidemics in the past, the genetic determinants of the virulence and transmissibility of influenza B viruses have not been studied in depth. In fact, the limited known host range of the influenza B virus and the paucity of available animal models have precluded investigation of influenza B pathogenicity and transmission factors, as well as the evaluation of antiviral compounds and vaccine efficacies in animals. In general, mice have been considered a good mammalian model for investigating pathogenic mechanisms and host range determinants of influenza viruses^11^. In this regard, mouse adaptation via serial passage in the lungs has often been used to study influenza A virulence factors in mice^12^,^13^,^14^,^15^,^16^. In addition, ferrets are a suitable small animal model with which to study influenza infection because they manifest flu-like symptoms and immune responses similar to those observed in humans^17^,^18^,^19^,^20^,^21^. While ferrets are used routinely to study the transmission of influenza A viruses^22^,^23^,^24^,^25^,^26^, prior to this study, their utility for influenza B virus transmission studies had not been established. Limited previous studies have shown that ferrets infected with B/Victoria-like and B/Yamagata-like viruses display mild clinical signs of infection and have lower elevations in core body temperature than do animals infected with influenza A viruses^27^; however, ferret-to-ferret transmission of influenza B viruses has not been evaluated to date.

In this study, we provide insight into the mammalian adaptation of influenza B viruses through molecular modifications that increase pathogenicity. Furthermore, we report that mouse-adapted influenza B viruses not only replicate in the upper respiratory tract of ferrets, but are also transmitted efficiently from infected to naïve animals by both direct and aerosol contact. Our results suggest that mutations in multiple genes encoding different viral proteins are associated with the growth and virulence of influenza B viruses.

## Results

### Genetic characterization of mouse-adapted B/Florida/04/06 influenza virus

To generate the mouse-adapted (MA) influenza B strain, B/Florida/04/06 was serially passaged in the lungs every three dpi until high virulence with 100% mortality was observed in BALB/c mice. After seventeen passages, infection with the B/Florida/ma04/06 virus resulted in severe influenza-like symptoms with 100% mortality in mice at 7 dpi. To investigate the molecular changes that occurred during the mouse adaptation of the virus, the P0 (B/Florida/04/05), three intermediate-passage viruses (P5, P9, and P12), and the seventeenth passage virus P17 (MA) were isolated by plaque purification, and the full-length sequences of each virus were analyzed. Compared with the P0 strain, the P17 (MA) strain possessed a total of seven nucleotide changes in four of the eight gene segments and five of the ten genes; these changes resulted in five amino acid mutations (Table 1). These amino acid mutations developed sequentially during the mouse adaptation process (Table 2). The first mutation that was observed was in the HA gene (D424G) in the P5 (MA_HA_) viruses (three of eight plaques). The second and third mutations occurred simultaneously in M1_N240T_ and BM2_N14S_ in the P9 (MA_M_) viruses, while six of eight plaques lost the HA gene (D424G) mutation. Increased virulence with 80% mortality was observed for P12 (MA_HA/M_), and full-length sequencing revealed that the P12 (MA_HA/M_) virus had all three of the mutations that were detected in the P5 and P9 viruses (HA_D424G_, M1_N240T_, and BM2 _N14S_). Notably, the fully mouse-adapted P17 (MA) virus expressed the three aforementioned mutations together with an additional two mutations in the NP_K294N_ and PA_V609I_ proteins. Together, these results demonstrate that mutations occurred sequentially during the mouse lung-to-lung passage of the B/Florida/04/06 virus and suggest that multiple genetic mutations might be required for the acquisition of high virulence by influenza B in mice.

### Growth characteristics of mouse-adapted influenza B viruses *in vitro*

**Figure 1:**
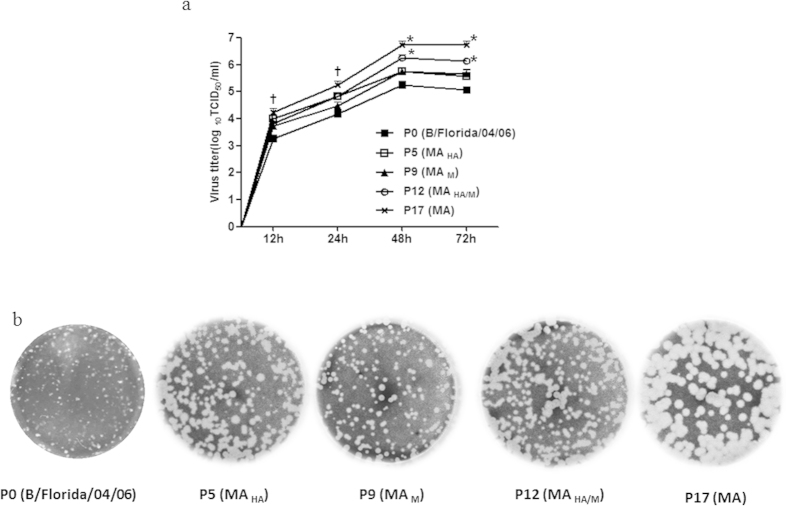
Replication kinetics of mouse-adapted virus. (**A**) Cells were infected with four influenza B viruses and mouse-adapted viruses at a multiplicity of infection^55^ of 0.001. Cell culture supernatants were collected at 12, 24, 48, and 72 hours post infection, and viral titers were measured in units of log_10_ TCID_50_/mL. **(B)** Determination of plaque phenotype. *indicates P < 0.0001, and †indicates P < 0.001 compared with the P0 (B/Florida/04/06). The error bars show the standard errors of the mean (SEMs), which were derived from three independent experiments.

To investigate whether sequential mutations of the B/Florida/04/06 virus affect its growth kinetics, each plaque-purified representative mutant, P0, P5 (MA_HA_), P9 (MA_M_), P12 (MA_HA/M_), and P17(MA), was inoculated onto MDCK cell monolayers, and the growth characteristics at 12, 24, 48, and 72 hours post infection (hpi) were assessed. The P0 virus reached a peak titer (5.0 log_10_ TCID_50_/mL) at 48 hpi, while the P17 MA virus showed a significantly higher peak viral titer (6.25 log_10_ TCID_50_/mL) than did the P0 virus (P < 0.001) at 48 hpi (Fig. 1A). Although the P5 (MA_HA_) and P9 (MA_M_) viruses did not display significantly higher peak titers in infected MDCK cells compared with the P0 virus, the P12 (MA_HA/M_) virus displayed significantly greater peak viral titers at 48 hpi (P < 0.001; Fig. 1A). These results clearly demonstrate that the serially passaged, mouse-adapted viruses have a considerable growth advantage *in vitro*.

Next, we compared the phenotypes of plaques formed in MDCK cells for each mouse-adapted virus. The P0 virus formed homogeneous, small, punctiform plaques (mean size, 1.0 ± 0.13 mm). During the course of mouse adaptation, the plaque sizes of each intermediate virus (P5, P9, and P12) became larger and more heterogeneous in size, ranging from ~1.5 mm to ~2.3 mm. Strikingly, the P17 (MA) virus produced large, homogeneous, circular plaques with an average size of 3.5 ± 0.11 mm (Fig. 1B). These results indicate that the P17 (MA) virus was homogeneous and readily replicated in MDCK cells.

### Pathogenicity of mouse-adapted influenza B viruses *in vivo*

**Figure 2:**
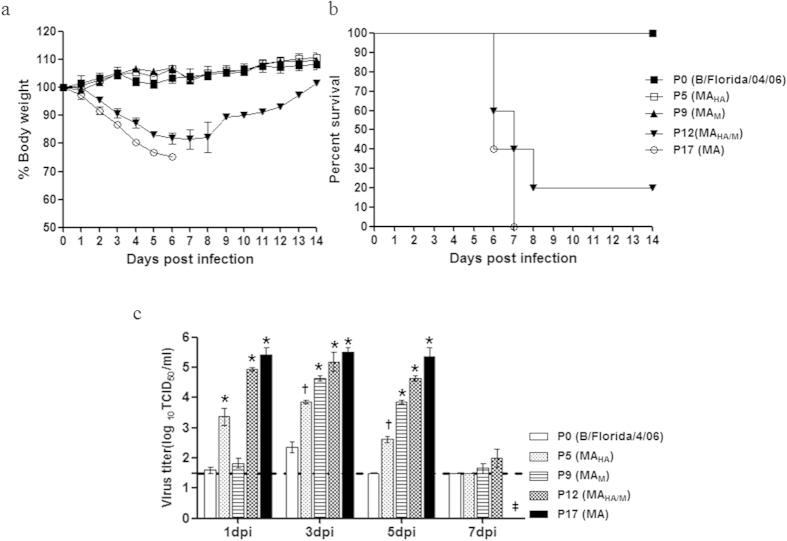
Pathogenicities of mouse-adapted influenza B viruses in mice. All groups of mice were observed for 14 days for **(A)** morbidity and **(B)** mortality. **(C)** Virus growth in mouse lungs. Five groups were infected intranasally with 6.0 log_10_ TCID_50_ of each virus. Lung virus titers on days 1, 3, 5, and 7 dpi are expressed as log_10_ TCID_50_ per gram of tissue. *indicates P < 0.0001, and † indicates P < 0.001 compared with the P0 (B/Florida/04/06); mouse death is indicated by ‡. The lower limit of detection (1.5 log_10_ TCID_50_) is represented by the dotted line.

To determine the pathogenicities of the mouse-adapted influenza B viruses in mice, we infected ten mice per group with each MA influenza B strain and monitored survival and weight loss for 14 dpi. The three cohorts of mice infected with P0 (parental virus), P5 (MA_HA_), and P9 (MA_M_) demonstrated 100% survival for 14 dpi, a very modest body weight loss (<1%), and no clinical symptoms (Fig. 2A). Mice infected with the P12 (MA_HA/M_) virus exhibited up to a 20% reduction in body weight by 8 dpi, and only two mice survived the infection (20% survival rate; Fig. 2B). In contrast, all P17 (MA)-infected mice became severely ill, lost body weight starting at 2 dpi (>25% from baseline), and eventually died or were humanly euthanized by 7 dpi (0% survival). These results suggest that mutations in the P12 (MA_HA/M_) and P17 (MA) viruses contribute to viral pathogenicity in mice.

We next assessed the viral replication kinetics in mouse lungs for each MA influenza B mutant to determine the association between lung viral titers and virulence. The parental virus (P0) could be recovered from mouse lungs until 5 dpi and reached peak titers (2.5 log_10_ TCID_50_/g) at 3 dpi. Although the P5 (MA_HA_) virus showed higher lung viral titers (3.5 log_10_ TCID_50_/g) than did P9 (MA_M_) at 1 dpi, the P9 (MA_M_) virus displayed greater viral titers compared with P5 (MA_HA_) at 3 and 5 dpi (Fig. 2C). However, the P12 (MA_HA/M_) virus had higher lung viral titers compared with those in the P0, P5 (MA_HA_), and P9 (MA_M_) groups until 7 dpi (5.25 log_10_ TCID_50_/mL peak titer). Finally, the P17 (MA) mutant-infected mice showed the highest viral lung titers until 5 dpi (5.5 TCID_50_/mL peak titer), and all mice succumbed to infection by 7 dpi (Fig. 2). These data suggest that the P12 (MA_HA/M_) and P17 (MA) viruses replicate efficiently in mouse lungs and that the P17 (MA) virus is better adapted to mouse lungs than the parental and intermediate viruses, as evidenced by its improved growth kinetics.

### Analysis of proinflammatory cytokine expression levels

**Figure 3:**
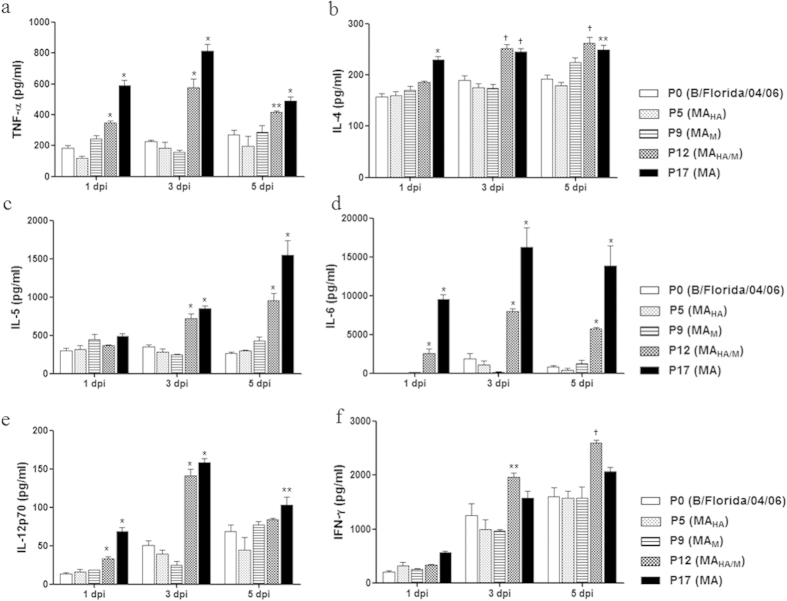
Cytokine expression associated with mouse-adapted influenza B virus. The mice were euthanized (n = 5 per each time point), and lung BALF was harvested. Cytokine production in lung BALF was analyzed, including the levels of TNF-α, IL-4, IL-5, IL-6, IL-12p70, and IFN-γ, by BioPlex analysis. Statistical significance compared to mice infected with the P0 (B/Florida/04/06) virus was determined by the *t*-test. *indicates P < 0.0001 and † indicates P < 0.001 compared with the P0 (B/Florida/04/06).

The dysregulation of proinflammatory cytokines, which is sometimes referred to as a cytokine storm, is associated with the pathogenesis of severe influenza virus infections^35^,^36^. Therefore, we investigated whether the infection of mice with mouse-adapted viruses results in cytokine responses related to pathogenicity in influenza B virus infections. As shown in Fig. 3, levels of the proinflammatory cytokines TNF-α, IL-4, IL-5 and IL-6 were higher in BALF from the P12 (MA_HA/M_) and P17 (MA) virus-infected mice relative to mice infected with the other viruses from 3dpi (p < 0.0001) through 5 dpi (p < 0.001; Fig. 3A–D). The levels of IL-12p70 were also elevated in the P12- (MA_HA/M_) and P17 (MA)-infected mice from 1dpi to 3 dpi compared with the P0 virus-infected mice; however, by 5dpi the levels of IL-12p70 decreased to levels comparable to those observed for the other groups (Fig. 3E). Only the P12 (MA_HA/M_)-infected group showed a significant upregulation of IFN-γ compared with the P0 virus-infected mice from 3 dpi (p < 0.05) to 5dpi (p < 0.001; Fig. 3F). These data suggest that the virulence of each mouse-adapted virus was closely correlated with the elevated proinflammatory cytokine expression levels observed in the lungs of infected mice.

### Replication and transmission of mouse-adapted influenza B viruses in ferrets

**Figure 4:**
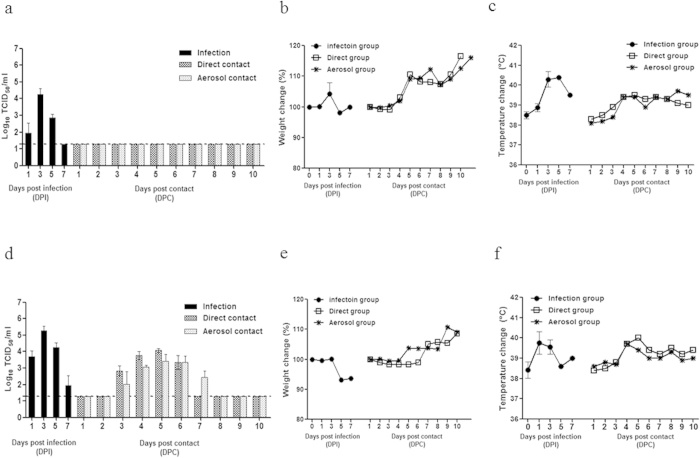
Weight loss, body temperature, and viral shedding in ferrets inoculated with mouse-adapted influenza virus. Two groups of ferrets (n = 12/group) were inoculated intranasally with 10^6^ TCID_50_ of virus. (**A**–**C**) P0 (B/Florida/04/06) and (D, E, and F) P17 (MA) virus. (**A**,**D**) Viral shedding from the nasal wash samples of inoculated ferrets. (**B**,**E**) Ferret weight changes during the mouse-adapted virus transmission experiments. Weight loss of inoculated animals is represented as a percentage of body weight. (**C**,**F**) Temperature change in inoculated animals is represented as a °C. After 24 h, each two inoculated animals in each group were individually paired and cohoused with direct contact ferrets (n = 2) and indirect aerosol contact ferrets (n = 2). The daily weights and temperature changes (as percentages) of each ferret were compared with the weights measured prior to the initiation of the experiments. Viral replication was monitored by titrating the viruses in the nasal washes that were collected every other day from both the inoculated (Left) and contact-exposed (Right) ferrets. The limit of detection was 1.5 log_10_TCID_50_/mL and is indicated by the dotted line for each representation.

Although the ferret is the most relevant small-animal model for studying human influenza A virus infections, a preferred transmission model for recent influenza B virus strains has not yet been described. To determine whether the P17 (MA) virus is transmissible as well as pathogenic in a ferret model, groups of ferrets were inoculated with each of virus and 24 h later, these ferrets were caged with or adjacent to naïve ferrets. The parental virus (P0) grew moderately well in the upper respiratory tract of the infected ferrets, reaching a mean peak titer of 4.75 log_10_ TCID_50_/mL at 3 dpi, and the virus was recovered in nasal washes until 5 dpi (Fig. 4A). There were no clinical signs of influenza infection in the ferrets infected with P0 (B/Florida/04/06) (<2% weight loss; Fig. 4A,B). The P17 (MA)-infected ferrets exhibited high viral titers (>10 times greater than that observed with P0) at all time points in the upper respiratory tract, with maximum nasal wash titers of 5.5 log_10_ TCID_50_/mL observed at 3 dpi. In addition, all ferrets inoculated with the P17 (MA) virus showed mild signs of influenza, marked by infrequent sneezing, a 2–8% reduction in mean body weight, and temperature elevations of up to 2.5 °C between 3 and 5 dpi (Fig. 4D–F).

**Figure 5:**
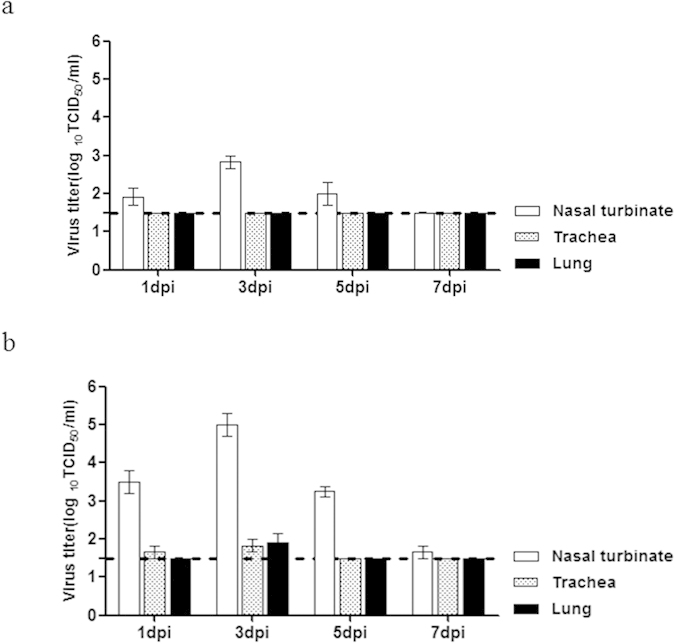
Mouse-adapted influenza B virus replication in the respiratory organs of ferrets. Ferrets (n = 2/day) were euthanized for virus titration on days 1, 3, 5, and 7 after infection. **(A)** P0 (B/Florida/04/06). **(B)** P17 (MA) virus. The virus titers in the nasal turbinates, trachea, and lung were determined in terms of the TCID_50_ in MDCK cells. Data are means ± SM of virus titration from three independent experiments. Data are representative of three independent experiments. The lower limit of detection (1.5 log_10_TCID_50_) is indicated by the dotted line.

Notably, only the P17 (MA) virus was readily transmitted to naïve ferrets via both direct contact and aerosol contact, as shown by the viral titers at 3dpc (4.0 log_10_ TCID_50_/mL at 5dpc; Fig. 4D). In contrast, no virus was recovered from the ferrets exposed either directly or through aerosol contact to P0 virus-infected ferrets (Fig. 4A). Furthermore, both direct and aerosol contact with P17 (MA) virus-infected ferrets resulted in high HI titers (160 HI GMT for direct contact and 40 HI GMT for aerosol contact) at 17 dpc (Table 3). Therefore, the P17 (MA) virus both maintained pathogenicity and exhibited enhanced airborne droplet transmission. A comparison of the viral titers in the ferret respiratory organs between the P0 (parent) and P17 (MA) viruses revealed increased replication of the P17 (MA) virus (up to 5.25 log_10_ TCID_50_/mL in the nasal turbinate of the inoculated ferrets), which was detected until 7 dpi. In contrast, infection with the P0 virus resulted in significantly lower viral titers at all time points (P < 0.05; Fig. 5). In addition, only the P17 (MA) virus was recovered from the trachea and lungs of infected ferrets.

**Figure 6:**
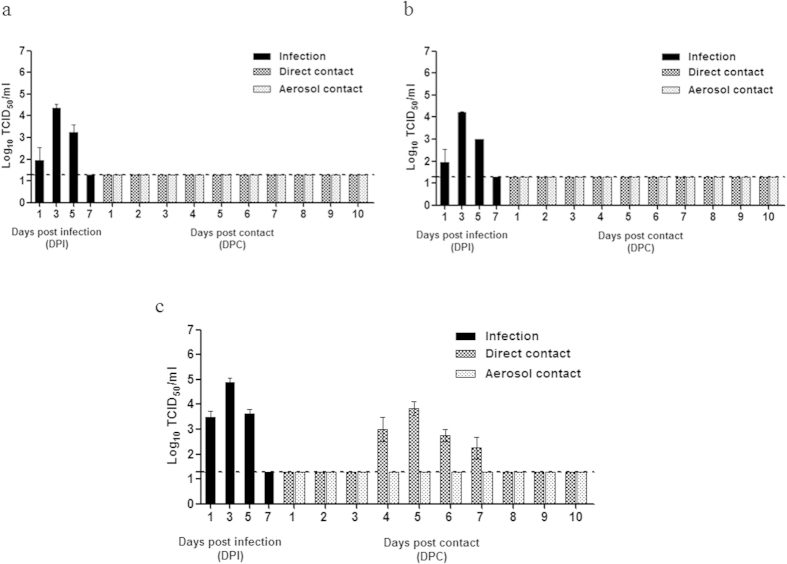
Transmission of mouse-adapted intermediate-passage influenza viruses in ferrets. Three groups of ferrets (n = 12/group) were inoculated intranasally with 10^6^ TCID_50_ of the following viruses: **(A)** P5 (MA_HA_), **(B)** P9 (MA_M_), and **(C)** P12 (MA_HA/M_). After 24 h, each two inoculated animals in each group were individually paired and cohoused with direct contact ferrets (n = 2) and indirect aerosol contact ferrets (n = 2). Viral replication was monitored by the titration of nasal washes collected every other day from both the inoculated (Left) and contact (Right) animals. The limit of detection was 1.5 log_10_TCID_50_/mL and is indicated by the dotted line.

**Figure 7:**
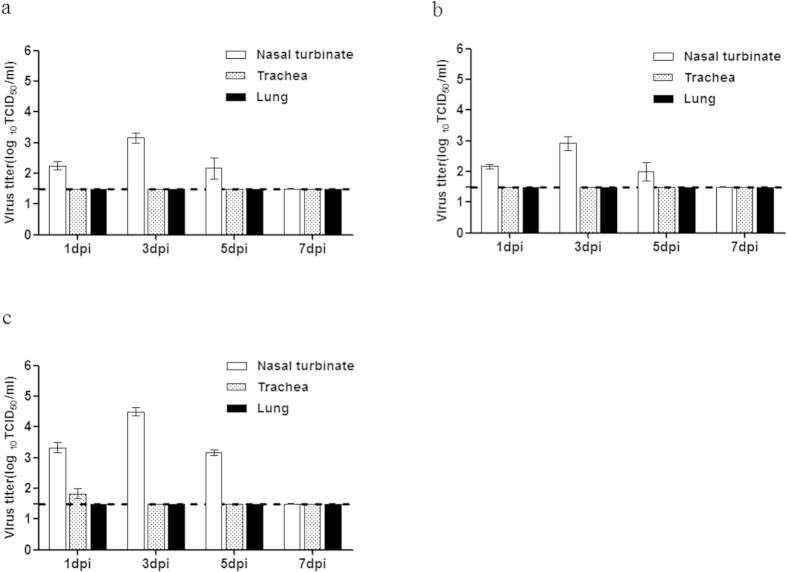
Mouse-adapted, intermediate-passage influenza virus replication in respiratory organs. Ferrets (n = 2/day) were euthanized on days 1, 3, 5, and 7 after infection for titration of the following viruses: (**A**) P5 (MA_HA_), (**B**) P9 (MA_M_), and (**C**) P12 (MA_HA/M_) viruses. The virus titers in the nasal turbinates, trachea, and lung were determined by measuring in terms of the TCID_50_ in MDCK cells. Data are representative of three independent experiments. The lower limit of detection (1.5 log_10_TCID_50_) is indicated by the dotted line.

Because the P17 (MA) virus exhibited efficient airborne droplet transmission to ferrets housed in adjacent cages, the remaining three intermediate mutants (P5 (MA_HA_), P9 (MA_M_), and P12 (MA_HA/M_)) were also assessed to determine which gene(s) or mutation(s) play critical roles in this process. We found that the P5 (MA_HA_) and P9 (MA_M_) viruses had viral replication properties similar to those of the wild-type P0 (parent) virus and failed to be transmitted either directly or through aerosol contact (Fig. 6A,B). Notably, the P12 (MA_HA/M_) virus showed increased viral peak titers (4.75 log_10_ TCID_50_/mL) compared to those of the wild-type P0 (parent), P5 (MA_HA_), and P9 (MA_M_) viruses and was transmitted to ferrets through direct contact, but not through aerosol contact (Fig. 6C). Only direct contact with the P12 (MA_HA/M_) virus-infected ferrets resulted in a modest serum HI titer (40 HI GMT) on day 17 dpc (Table 3). Moreover, viral titers approximately five to ten times higher (3.25 to 4.75 log_10_ TCID_50_/g) were observed in P12 (MA_HA/M_) virus-infected nasal turbinates compared with those infected with the P5 (MA_HA_) and P9 (MA_M_) viruses, and these elevated levels persisted until 5 dpi (Fig. 7). In contrast, no virus was detected in the lungs or trachea, with the exception of infection with the P12 (MA_HA/M_) virus at 1 dpi, suggesting that the ability of each intermediate mutant to replicate in the respiratory tracts of infected ferrets was impaired compared with the P17 (MA) variant. Taken together, our results indicate that although the clinical symptoms caused by the P17 (MA) virus were not significantly different from those caused by the wild-type P0 (parent) virus, mutations in various genes generated during mouse adaptation collectively contributed to the transmissibility of the influenza B virus in the ferret model. Furthermore, the finding that the P17 (MA) virus replicates efficiently in the upper respiratory tract supports the finding that this virus is transmitted efficiently between ferrets, even by indirect means.

### Polymerase activity is enhanced in a mouse-adapted influenza B virus

**Figure 8:**
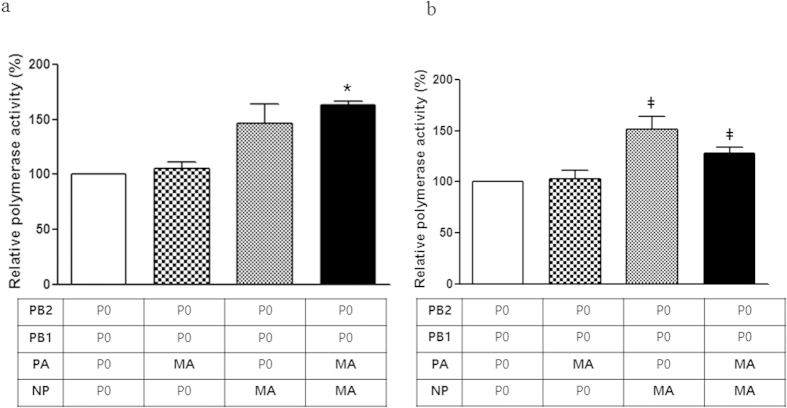
Enhanced polymerase activity of mouse-adapted influenza B virus. The polymerase activities of reconstituted RNP complexes composed of the PB2, PB1, PA, and NP plasmids of the virus strains P0 (B/Florida/04/06) and P17 (MA). The indicated combinations and single-point mutants are shown. Note that PB2 and PB1 are the same in all complexes because no mutation was observed in this gene. The luciferase activity values are the mean values of at least three assays; **(A)** 33 °C and **(B)** 37 °C. *indicates P < 0.0001, and ‡indicates P < 0.01 compared with the parent virus (P0).

To determine to what extent the PA and NP mutations were responsible for the high titer of the P17 (MA) virus observed in the upper respiratory tract (turbinate), we assessed polymerase activity using a luciferase reporter assay^34^. The PA (V609I) and/or NP (K294N) mutations were incorporated into parental virus, individually or simultaneously, using site-directed mutagenesis. At 33 °C, a temperature representative of the upper respiratory tract, polymerase complexes with the individual PA and NP mutants showed approximately 105% and 146% polymerase activity, respectively, compared with that of the parent polymerase complex (Fig. 8). Notably, the simultaneous PA and NP mutants showed the highest polymerase activity (163%) out of all of the viral polymerase complexes tested at 33 °C (P < 0.0001)(Fig. 8). However, the polymerase activity of the simultaneous PA and NP mutant (124%) was lower than that of the single NP (294N) mutant (150%) at 37 °C. Nevertheless, these results indicate that the NP (294N) mutation increases polymerase activity at both 33 °C and 37 °C.

## Discussion

Viral adaptation to a specific host is considered to be one of the primary mechanisms underlying influenza virus evolution. The resulting zoonotic influenza viruses have the potential to become novel pandemic influenza strains that can spread throughout immunologically-compromised human populations^37^,^38^,^39^. Although the influenza B virus can induce severe disease and even death in human populations, most human influenza B virus strains are avirulent in mouse models, only transiently infecting mice producing low titers in the lungs.

In this study, we selected a recent influenza B vaccine strain (B/Florida/04/06) and generated a mouse-adapted, lethal B/Florida/ma04/06 virus. Of note, full-length sequencing of intermediate strains revealed the mutations that were observed in four genes (the HA (P5 and P12), M1 (P9 and P12), M2 (P9 and P12), NP (P17), and PA (P17) genes; Table 2) accumulated serially. However, increased virulence was observed between the P12 (MA_HA/M_) and P17 (MA) passages of B/Florida/04/06. A relatively high degree of virulence (80% case fatality rate and an MLD_50_ of 6.5 TCID_50_ was first observed in the P12 (HA_N424G_, M1_N240V_, and BM2_N107S_) virus, and the P17 (MA) mutant strain showed 100% mortality with a low MLD_50_ of 5.5 TCID_50_. These results suggest that multigenic factors are associated with the influenza B virus virulence and demonstrate that the HA, NP, M, and PA genes play important roles in the virulence of B/Florida/04/06.

Previous studies have focused on the roles of the M1 and NS1 proteins in increased virulence following the adaptation of influenza viruses in mouse lungs^32^,^40^,^41^,^42^. Similarly, McCullers *et al.* showed that the serial passage of an initially avirulent influenza B virus (B/Memphis/12/97) results in the selection of a lethal mouse-adapted strain, and suggested a critical role for a single amino acid mutation (N221S) in the C-terminal domain of the M1 gene product^40^. However, no mutation was commonly found between the current study and the previous B/Memphis/12/97 study. In fact, only one similar mutation (N240V) in the C-terminal domain of the M1 gene was acquired as early as P9 in the current study, but *in vivo* experiments revealed no critical role of this mutation in increased virulence (Fig. 2). The influenza B virus NS1 has been shown to bind to the interferon-stimulated gene 15 protein (ISG15) and subsequently to prevent the ISGylation of target proteins, thereby blocking downstream antiviral effects^43^,^44^,^45^. Of note, we did not detect any NS1 mutations in the B/Florida/04/06 strain until passage 17. Interestingly, we identified a consensus change in the HA gene (HA_N424G_) in the P5 and P12 viruses. A single HA mutation in the P5 virus was associated with increased viral growth kinetics in infected mouse lungs at early time points, although there was no increase in body weight loss or mortality caused by this virus (Fig. 2). In contrast, a virus with a combination of mutations in M1, BM2, and HA (P12, MA_HA/M_) caused significantly more body weight loss and mortality and enhanced viral growth kinetics compared with the parental strain. Subsequently, additional NP and PA mutations were found only in the fully mouse-adapted, lethal B/Florida/ma04/06 P17 (MA) strain. These results suggest that the HA mutation may increase the susceptibility of mice to the B/Florida/04/06 virus and rather than a single amino acid mutation, it is the combination of this HA mutation with mutations in internal genes (M1, BM2, NP, and PA genes) that is critical for high virulence in mice.

Inflammatory cytokines and chemokines are produced during influenza virus infections^46^,^47^, which is the first consequence of the activation of innate immune cells. However, the multiple functions of cytokines can be either beneficial or detrimental to virus-infected hosts. Previous studies have suggested that IL-6 is an early inflammatory marker, and its level has been shown to be well-correlated with infection severity^48^ and a Th2 cytokine (IL-4 and IL-5)-mediated delay in viral clearance^49^,^50^. In addition, IL-12 has been reported to contribute to early NK cell-dependent IFN-γ production and to inhibit early influenza virus replication in mice^51^. In this study, the evaluation of various mouse-adapted B/Florida/04/06 virus infections revealed notable patterns in cytokine expression in the P12 (MA_HA/M_) and P17 (MA) groups relative to the parental (P0) group (Fig. 3). We found that proinflammatory cytokine (IL-5, IL-4, and IL-6) production was significantly increased later in the P17 (MA) viral infection process (at 5 dpi) compared with the parent (P0) virus infection. In addition, the immunoregulatory interleukin IL-12p70, which is involved in the differentiation of naïve T cells into Th1 cells, was robustly induced early in the course of infection (at 1 and 3 dpi) in the P12 (MA_HA/M_) and P17 (MA) groups. This increased expression of inflammatory cytokines may be predominantly reflective of the enhanced virulence of these viruses in infected mice. Thus, the induction of proinflammatory cytokines was positively correlated with viral loads in the lung at the early stages of infection, and their prolonged expression may be the cause of the high mortality.

Ferrets are a suitable model in which to study influenza infection because they manifest human flu-like symptoms and immune responses^17^,^52^. In fact, the utility of the ferret model for studying the clinical and pathogenic features of influenza B virus infection have been demonstrated previously. Stephen S. H. *et al.* found that certain influenza B strains, such as B/Brisbane/60/2008, establish persistent lower respiratory tract infections in ferrets, suggesting that the influenza B virus may cause moderate to severe clinical illness in these animals^53^. In this study, we demonstrate that mouse-adapted B/Florida/04/06 viruses not only replicate in the upper and lower respiratory tracts of ferrets but they were also efficiently transmitted from infected to naïve animals under both contact and aerosol droplet transmission conditions (Fig. 4). Interestingly, only the P17 (MA) virus transmitted readily to direct contact- and aerosol contact-naïve ferrets at 3 dpc, while no virus was recovered from either contact group exposed to the P0 virus-infected ferrets. To our knowledge, this is the first report of ferret-to-ferret aerosol droplet transmission of influenza B virus.

In addition, the results with three intermediate passage mutants (P5, P9, and P12) suggest that the limited replication of the influenza B virus in the upper respiratory tract could explain why influenza B virus transmission has not been previously documented in ferrets. Nevertheless, we hypothesize that the P12 virus (with the HA_N424G_, M1_N240V_, and BM2_N14S_ mutations) possesses an increased ability to replicate in ferrets and that the mutations present in this virus are responsible for its ability to transmit to ferrets via direct contact. Furthermore, additional NP and PA gene mutations, which are closely associated with polymerase activity, are crucial for efficient viral replication in the upper respiratory tract and for aerosol transmission in the ferret model, as observed in P17 (MA) virus-infected ferrets. Interestingly, the NP and PA genes are core components of the viral ribonucleoprotein (vRNP) complex of influenza viruses and are also known to harbor host range determinants^54^,^55^. In particular, the PB2 and PA polymerase subunits of influenza A viruses are essential for the efficient replication of avian influenza viruses in mammals^14^,^56^,^57^ and also affect the host range and pathogenicity of avian influenza viruses^58^. A recent study has found that an arginine to lysine substitution at position 185 of an H5N1 virus PA protein significantly affects virulence and pathogenicity in mice^59^. Interestingly, an NP protein mutation similar to that observed in the B/Florida/ma04/06 virus (K294N) has been identified in the avian A/H7N1 NP protein (V284M) after passage in ferrets, resulting in the acquisition of the ability to transmit via aerosols. This mutation is in the C-terminal PB2 interaction domain and occurs in parallel with a mutation in the PB2 N-terminal domain (PB2_T81I_)^60^, indicating that together these mutations may enhance polymerase activity. Further, a polymerase activity assay indicated that a point mutation at the NP 294N residue is sufficient to enhance polymerase activity in mammalian cells at both temperatures tested (33 °C and 37 °C), while a PA mutation (609I) is additionally required for high polymerase activity at a temperature representative of the upper respiratory tract, such as that in the nasal turbinate. Therefore, we hypothesize that the increased polymerase activity of the P17 (MA) mutant, which contains both the PA and NP mutations, led to the high viral titer observed in the nasal turbinate and that this may have contributed to the elevated rate of transmission to ferrets exposed via direct and aerosol contact in our study.

In conclusion, this study demonstrates that mouse adaptation is a useful strategy for efficiently generating influenza B viruses for the infection of various mammalian hosts. In addition, multigenic virulence factors, including mutations in HA, M, and the vRNP complex, are required for efficient replication in the upper respiratory tract, which contributes to transmission of type B influenza viruses in ferrets. Thus, these results provide a foundation for the investigation of influenza B virus transmission in an animal model, and also emphasize the persistent surveillance for containment purposes and reduce opportunities for further genetic evolution. Moreover, this model may also prove useful for investigating influenza B virus biology, including virulence markers, as well as for vaccine development, vaccine efficacy, and antiviral compound testing in *in vivo* experiments.

## Materials and Methods

### Cells

Madin-Darby canine kidney (MDCK) cells obtained from the American Type Culture Collection (ATCC) were maintained in Eagle’s minimal essential medium (EMEM; Lonza, Inc., Allendale, NJ) supplemented with 10% fetal bovine serum (Lonza, Inc.), 1% penicillin/streptomycin (Gibco-Invitrogen, Inc., Carlsbad, CA), and 1% nonessential amino acids (Gibco-Invitrogen, Inc.). 293T human embryonic kidney cells were grown in Dulbecco’s modified Eagle’s medium containing 10% FBS and 1% penicillin/streptomycin (Gibco-Invitrogen, Inc., Carlsbad, CA).

### Viruses

Influenza virus B/Florida/04/2006 (Yamagata lineage) was obtained from the Green Cross Corporation and passaged in mice. It was serially passaged 17 times in mice until a mouse-adapted, highly virulent strain was generated, which was termed the mouse-adapted (P17 (MA)) variant. The parent virus (P0), 3 intermediate passages (P5, P9, and P12), and the 17th passage (P17 (MA)) were isolated by plaque purification, as described below, and were selected based on sequence differences relative to the parental wild-type virus.

### Plaque purification and selection

To isolate single-phenotype viruses that cause mortality at rates similar to those observed with the mouse-adapted virus, we plaque-purified lung isolates of the virulent mouse-adapted strain from MDCK cells, as described previously^28^. Briefly, supernatants of lung tissue homogenates were serially diluted 10-fold in appropriate media. MDCK cells were infected with the diluted samples in six-well plates. After 1 h of incubation, the cells were washed with PBS and overlaid with a 0.7%-agarose-medium mixture containing 1 μg/mL of L-1-tosylamide-2-phenylmethyl chloromethyl ketone (TPCK)-treated trypsin. Seventy-two hours later, eight single-plaque colonies were selected from each plate, resuspended in medium, and used to infect MDCK cells. After 48 h of incubation, viruses were harvested, and the 50% tissue culture infectious dose (TCID_50_) of each strain was calculated by the method of Reed and Muench^29^. After the colonies were selected from plates, the MDCK cells on the plates were fixed with 4% ice-cold formalin and stained with 1% crystal violet in order to count and measure plaques. The average plaque size was calculated as an arithmetic mean of the sizes of fifteen or more individual plaques. Viruses that formed plaques of ≤1.0 mm, 1.0–1.9 mm or ≥2.0 mm in diameter on average were designated as having small-size, medium-size or large-size plaque phenotypes, respectively^30^.

### Genomic sequencing and phylogenetic analyses

Viral RNA was extracted from cell culture isolates using an RNeasy Mini Kit (Qiagen, Valencia, CA). RT-PCR and full-genome sequencing were performed under standard conditions using influenza-specific primers as previously described^31^,^32^. DNA sequences were compiled and edited using the DNAStar Lasergene sequence analysis software package version 5.0 (Madison, WI).

### *In vitro* replication assays

MDCK cells were prepared in 6-well plates and allowed to grow for 24 h, after which they were infected with B/Florida/04/2006 (P0), three intermediate-passage viruses (P5, P9, and P12), and one 17th passage (P17 (MA))virus at a multiplicity of infection of 0.001. Infected cells were incubated at 33 °C in appropriate medium containing 0.2% bovine serum albumin and TPCK-treated trypsin. Supernatants were collected at 12, 24, 48, and 72 h post infection (p.i.), and the viruses were titrated (log_10_ TCID_50_) in MDCK cells by the method of Reed and Muench^29^.

### Studies in mice

Five groups of BALB/c (H-2d) mice (5-week-old females weighing ≥18 g/mouse; Samtaco, Seoul, Republic of Korea) were anesthetized with an intraperitoneal injection of a Zoletil/xylazine mixture (Zoletil 50^®^, 80 mg/kg, Virbac, France; Rompun^®^, 20 mg/kg, Bayer HealthCare, Germany) and inoculated intranasally (i.n.) with 6.0 log_10_ TCID_50_ of virus. The mice (n = 10/group) were monitored for weight loss and mortality daily for fourteen days. A mouse was euthanized and, for the purpose of data analysis, was considered to have died if its body weight was reduced by >25% compared with its original body weight. The experimental animals were used and handled according to the principles described in the Guide for the Health and Conditions of Mice^33^. Each group of mice (n = 5) were euthanized on days 1, 3, 5, and 7 p.i. Lungs were collected and homogenized (1 g/mL) in cold phosphate-buffered saline (PBS) containing antibiotics (0.1% penicillin/streptomycin; Gibco). The supernatants were serially diluted 10-fold and inoculated into MDCK cells for virus titration (log_10_ TCID_50_ per gram of tissue). After 72 h, hemagglutination assays were performed using 0.5% turkey red blood cells. To determine the 50% mouse lethal dose (MLD_50_) of the viruses, we inoculated groups of ten mice i.n. with 10-fold serial dilutions containing 10^1^ to 10^6^ TCID_50_ of virus in a 30 μL volume. The MLD_50_ was expressed in terms of log_10_ TCID_50_. All TCID_50_ and MLD_50_ calculations were performed according to Reed and Muench^29^.

### Studies in ferrets

Sixteen- to 18-week-old adult outbred female ferrets (*Mustela putorius furo*) weighing 0.5 to 0.8 kg (Wuxi Sangosho Pet Park Co., Wuxi, China) were tested for the absence of antibodies to currently circulating influenza viruses (H5, H7, H9, pH1N1, human seasonal H1N1 and H3N2, and all of the viruses used in this study) using hemagglutination inhibition (HI) assays. For pathogenesis and transmission experiments, 10^6.0^ TCID_50_ of the mouse-adapted influenza B viruses (P0, P5, P9, P12, and P17) in 1 mL of sterile PBS was instilled intranasally (i.n.) (500 μL/each nostril) into five groups of ferrets (n = 12/group) under anesthesia. After 24 h, inoculated animals in each group were individually paired and cohoused with direct contact ferrets (n = 2). For aerosol contact, naïve ferrets (n = 2) were housed in the other half of a cage containing inoculated ferrets, and the ferrets were separated by two stainless steel grids, allowing for airborne virus transmission but preventing transmission via direct contact. The ferrets were weighed and observed for clinical signs of infection daily for up to 11 days post infection (dpi). Nasal washes were collected every other day from the inoculated ferrets and daily from the contact ferrets for virus titration. Ferrets (n = 2) from each inoculated group were euthanized at 1, 3, 5, and 7 dpi, and the viral titers in the lungs, trachea, and nasal turbinate were assessed using the TCID_50_ method. All remaining inoculated and contact animals were euthanized at 18 dpi (17 days post contact, dpc), and their blood samples were tested for specific antibodies to a homologous virus with the HI assay using 0.5% turkey erythrocytes.

### BALF samples and cytokine detection by multiplex microbead immunoassay

Bronchoalveolar lavage fluid (BALF) samples were collected from mouse lungs at 1, 3, and 5 dpi or from uninfected control mouse lungs (five mice/group). The BALF samples were centrifuged at 12 000 rpm for 5 min at 4 °C, aliquoted, and stored at −70 °C until analysis. A multiplex biometric immunoassay using fluorescently dyed microspheres conjugated with monoclonal antibodies specific for target proteins was used for cytokine measurements according to the manufacturer’s instructions (Panomics Mouse Cytokine Assay; Affymetrix Inc., Fremont, CA). Levels of the following cytokines were measured: IL-4, IL-5, IL-6, IL-12p70, interferon-gamma (INF-γ), and tumor necrosis factor-alpha (TNF-α). Briefly, 20 μL of BALF was diluted 1:4 and incubated with antibody-coupled beads. Complexes were washed, incubated with a biotinylated detection antibody and labeled with streptavidin–phycoerythrin before cytokine concentrations were measured. Concentrated mouse recombinant cytokines were obtained from Panomics Laboratories. A range of 9350–25 000 pg/mL of recombinant cytokines was used to establish standard curves and to maximize the sensitivity and dynamic range of the assay. The cytokine levels were determined using a Luminex™ Instrumentation Systems multiplex array reader (Bio-Plex Workstation from Bio-Rad Laboratories). Cytokine concentrations were calculated using software provided by the manufacturer (Bio-Plex Manager Software).

### Luciferase mini-genome reporter assays

A luciferase reporter plasmid (pHW72-B/Luc) driven by a pol I transcription unit containing the non-coding region of the influenza B virus M gene was constructed. Luciferase activity was measured according to Salomon *et al.*^34^ to compare the activities of the recombinant viral polymerase complexes. 293T cells were prepared in 24-well plates 24 h before use and were transfected with 0.1 μg each of pHW72-B/*Luc*, pHW-B/2000-PB2, pHW-B/2000-PB1, pHW-B/2000-PA, pHW-B/2000-NP, and pCMV-β*-*galactosidase plasmids using the TransIT-LT1 transfection reagent as directed. After 4 h, the transfection medium was replaced with fresh DMEM (Gibco) with 5% FBS. After 24 h, the cells were washed with PBS and lysed for 30 min with 100 μL of lysis buffer (Promega). The cell lysates were then harvested, and luciferase activity was assayed in triplicate using a Promega Luciferase Assay System. The results were normalized to the β-galactosidase activity level of the cells.

### Statistical analyses

To assess significant differences in viral titers and cytokine expression levels, one-tailed unpaired Student’s *t*-tests were used. Welch’s correction was applied when variances were found to be inhomogeneous. All statistical analyses were performed using Prism 5 (GraphPad Software).

### Ethics statement

All animals were housed at an appropriate temperature under a standard light/dark cycle. The research protocols involving the use of all animals in this study were conducted in strict accordance with the animal handling policies mandated by the Guidelines for Animal Use and Care of the Korea Center for Disease Control (K-CDC) and were approved by the Medical Research Institute of Chungbuk National University in Korea (approval number CBNU-IRB-2012-GM01 and CBNUA-856-15-01). All of the experiments were progressed in an Enhanced Animal BioSafety Level 3 (ABSL3+) facility in Chungbuk National University permitted by the Center for Disease Control in the Republic of Korea.

## Additional Information

**How to cite this article**: Kim, E.-H. *et al.* Mouse adaptation of influenza B virus increases replication in the upper respiratory tract and results in droplet transmissibility in ferrets. *Sci. Rep.* **5**, 15940; doi: 10.1038/srep15940 (2015).

## Figures and Tables

**Table 1:** Nucleotide and amino acid changes in wild-type and mouse-adapted influenza virus B/Florida/04/2006.

Genesegment	Changed nucleotides^a^			Changed amino acids^b^		
	Position	Original	Mouse- adapted	Position	Original	Mouse-adapted
HA	951	T	A	317	P	—
	1271	A	G	424	D	G
M1^c^	719	A	C	240	N	T
BM2^c^	27	C	T	9	I	—
NP	40	A	G	14	N	S
	882	G	C	294	K	N
PA	1825	G	A	609	V	I

^a^Numbering of nucleotides is relative to B/Lee/40 and begins at the start of the non-coding region of the RNA segment.

^b^Numbering of amino acids is relative to B/Lee/40 and begins at the start of the coding region.

^c^RNA segment 7 encodes two polypeptides, M1 and BM2, using tandem cistrons.

**Table 2:** Sequences of amino acid changes during mouse adaptation.

Viral proteins(amino acid)	P0	P5	P9	P12	P17
HA (424)	Asp	Gly	—	Gly	Gly
M1 (240)	Asn	—	Thr	Thr	Thr
M2 (14)	Asn	—	Ser	Ser	Ser
NP (294)	Lys	—	—	—	Asn
PA (609)	Val	—	—	—	Ile

Note: P0, parent virus B/Florida/04/2006; P5, 5th mouse passage; P9, 9th mouse passage; P12, 12th mouse passage; and MA, mouse-adapted virus after 17 passages.

**Table 3:** Seroconversion associated with mouse-adapted influenza B viruses in ferrets exposed to direct and aerosol contact.

Virus	Direct contact	Aerosol contact
	Serum (HI GMT*)†	
P0 (Parent)	<10	<10
P5 (MA_HA_)	<10	<10
P9 (MA_M_)	<10	<10
P12 (MA_HA/M_)	40	<10
P17 (MA)	160	40

^†^The parent virus was used in HI assays; sera were collected at 17 dpc.

^*^Note: GMT, geometric mean titers.

## References

[b1] Palese P. & Shaw M. L. In In Fields Virology (ed Knipe D. & Howley P. M.) 1647–1689 (Lippincott Williams & Wilkins, 2007).

[b2] Ni F., Kondrashkina E. & Wang Q. Structural basis for the divergent evolution of influenza B virus hemagglutinin. Virology 446, 112–122 (2013). 24074573 10.1016/j.virol.2013.07.035 PMC3902124

[b3] Lin Y., Gregory V., Bennett M. & Hay A. Recent changes among human influenza viruses. Virus Res 103, 47–52 (2004). 15163487 10.1016/j.virusres.2004.02.011

[b4] Belshe R. B., Coelingh K., Ambrose C. S., Woo J. C. & Wu X. Efficacy of live attenuated influenza vaccine in children against influenza B viruses by lineage and antigenic similarity. Vaccine 28, 2149–2156 (2010). 20003926 10.1016/j.vaccine.2009.11.068

[b5] Ikonen N., Pyhälä R., Axelin T., Kleemola M. & Korpela H. Reappearance of influenza B/Victoria/2/87-lineage viruses: epidemic activity, genetic diversity and vaccination efficacy in the Finnish Defence Forces. Epidemiology and Infection 133, 263–271 (2005). 15816151 10.1017/s0950268804003462 PMC2870245

[b6] Control C. F. D. & Prevention. Influenza activity-United States and worldwide, 2007-08 season. MMWR. Morbidity and mortality weekly report 57, 692 (2008). 18583957

[b7] Reed C., Meltzer M. I., Finelli L. & Fiore A. Public health impact of including two lineages of influenza B in a quadrivalent seasonal influenza vaccine. Vaccine 30, 1993–1998 (2012). 22226861 10.1016/j.vaccine.2011.12.098

[b8] Chang C., New A., Taylor J. & Chiang H. Influenza virus isolations from dogs during a human epidemic in Taiwan. (DTIC Document, 1976). 977232

[b9] Osterhaus A., Rimmelzwaan G., Martina B., Bestebroer T. & Fouchier R. Influenza B virus in seals. Science 288, 1051–1053 (2000). 10807575 10.1126/science.288.5468.1051

[b10] Takátsy G, R. J. & Farkas E. Susceptibility of the domestic swine to influenza B virus. Acta Microbiol Acad Sci Hung. 14, 309–315 (1967). 5625091

[b11] Ward A. C. Virulence of influenza A virus for mouse lung. Virus Genes 14, 187–194 (1997). 9311563 10.1023/a:1007979709403

[b12] Ilyushina N. A. *et al.* Adaptation of pandemic H1N1 influenza viruses in mice. J Virol 84, 8607–8616 (2010). 20592084 10.1128/JVI.00159-10 PMC2918990

[b13] Ping J. *et al.* PB2 and hemagglutinin mutations are major determinants of host range and virulence in mouse-adapted influenza A virus. J Virol 84, 10606–10618 (2010). 20702632 10.1128/JVI.01187-10 PMC2950562

[b14] Czudai-Matwich V., Otte A., Matrosovich M., Gabriel G. & Klenk H.-D. PB2 Mutations D701N and S714R Promote Adaptation of an Influenza H5N1 Virus to a Mammalian Host. J Virol, JVI. 00422–00414 (2014). 10.1128/JVI.00422-14 PMC4136279 24899203

[b15] de Jong R. *et al.* Rapid emergence of a virulent PB2 E627K variant during adaptation of highly pathogenic avian influenza H7N7 virus to mice. Virology journal 10, 276 (2013). 24007444 10.1186/1743-422X-10-276 PMC3766704

[b16] Liu Q. *et al.* Virulence Determinants in the PB2 Gene 1 of a Mouse-Adapted H9N2 Virus. J Virol, JVI. 01775–01714 (2014). 10.1128/JVI.01775-14 PMC4301167 25339773

[b17] Banner D. & Kelvin A. A. The current state of H5N1 vaccines and the use of the ferret model for influenza therapeutic and prophylactic development. The Journal of Infection in Developing Countries 6, 465–469 (2012). 22706187 10.3855/jidc.2666

[b18] Hamelin M.-È. *et al.* Oseltamivir-resistant pandemic A/H1N1 virus is as virulent as its wild-type counterpart in mice and ferrets. PLoS Pathog 6, e1001015 (2010). 20661429 10.1371/journal.ppat.1001015 PMC2908621

[b19] Hause B. M. *et al.* Isolation of a novel swine influenza virus from Oklahoma in 2011 which is distantly related to human influenza C viruses. PLoS pathogens 9, e1003176 (2013). 23408893 10.1371/journal.ppat.1003176 PMC3567177

[b20] Huang S. S. *et al.* Comparative analyses of pandemic H1N1 and seasonal H1N1, H3N2, and influenza B infections depict distinct clinical pictures in ferrets. PLoS One 6, e27512 (2011). 22110664 10.1371/journal.pone.0027512 PMC3217968

[b21] León A. J. *et al.* Sequencing, annotation, and characterization of the influenza ferret infectome. Journal of virology 87, 1957–1966 (2013). 23236062 10.1128/JVI.02476-12 PMC3571481

[b22] Belser J. A. *et al.* Pathogenesis and transmission of avian influenza A (H7N9) virus in ferrets and mice. Nature (2013). 10.1038/nature12391 PMC7094885 23842497

[b23] Herfst S. *et al.* Airborne transmission of influenza A/H5N1 virus between ferrets. Science 336, 1534–1541 (2012). 22723413 10.1126/science.1213362 PMC4810786

[b24] Tellier R. Aerosol transmission of influenza A virus: a review of new studies. Journal of the Royal Society Interface, rsif20090302 (2009). 10.1098/rsif.2009.0302.focus PMC2843947 19773292

[b25] Wan H. *et al.* Replication and transmission of H9N2 influenza viruses in ferrets: evaluation of pandemic potential. PLoS One 3, e2923 (2008). 18698430 10.1371/journal.pone.0002923 PMC2500216

[b26] Zhu H. *et al.* Infectivity, transmission, and pathology of human-isolated H7N9 influenza virus in ferrets and pigs. Science 341, 183–186 (2013). 23704376 10.1126/science.1239844

[b27] Kim Y. H., Kim H. S., Cho S. H. & Seo S. H. Influenza B virus causes milder pathogenesis and weaker inflammatory responses in ferrets than influenza A virus. Viral immunology 22, 423–430 (2009). 19951179 10.1089/vim.2009.0045

[b28] Song M.-S. *et al.* The polymerase acidic protein gene of influenza a virus contributes to pathogenicity in a mouse model. J Virol 83, 12325–12335 (2009). 19793828 10.1128/JVI.01373-09 PMC2786751

[b29] Reed L. J. A. M., H. A simple method of estimating fifty percent endpoints. . The American Journal of Tropical Medicine and Hygiene 27, 493–499 (1938).

[b30] Lugovtsev V. Y., Vodeiko G. M., Strupczewski C. M., Ye Z. & Levandowski R. A. Generation of the influenza B viruses with improved growth phenotype by substitution of specific amino acids of hemagglutinin. Virology 365, 315–323 (2007). 17490701 10.1016/j.virol.2007.04.006

[b31] Hoffmann E. *et al.* Rescue of influenza B virus from eight plasmids. Proceedings of the National Academy of Sciences 99, 11411–11416 (2002). 10.1073/pnas.172393399 PMC123270 12172012

[b32] Jackson D., Elderfield R. A. & Barclay W. S. Molecular studies of influenza B virus in the reverse genetics era. Journal of General Virology 92, 1–17 (2011). 20926635 10.1099/vir.0.026187-0

[b33] Foltz C. J. & Ullman-Cullere M. Guidelines for assessing the health and condition of mice. RESOURCE 28 (1999). 10403450

[b34] Wunderlich K. *et al.* Identification of a PA-binding peptide with inhibitory activity against influenza A and B virus replication. PLoS One. 10, e7517 (2009). 19841738 10.1371/journal.pone.0007517 PMC2759517

[b35] Hayden F. G. *et al.* Local and systemic cytokine responses during experimental human influenza A virus infection. Relation to symptom formation and host defense. Journal of Clinical Investigation 101, 643 (1998). 9449698 10.1172/JCI1355 PMC508608

[b36] Us D. Cytokine storm in avian influenza. Mikrobiyoloji bulteni 42, 365–380 (2008). 18697437

[b37] Chen H. *et al.* Polygenic virulence factors involved in pathogenesis of 1997 Hong Kong H5N1 influenza viruses in mice. Virus Res 128, 159–163 (2007). 17521765 10.1016/j.virusres.2007.04.017

[b38] Chin P. *et al.* Molecular evolution of H6 influenza viruses from poultry in Southeastern China: prevalence of H6N1 influenza viruses possessing seven A/Hong Kong/156/97 (H5N1)-like genes in poultry. J Virol 76, 507–516 (2002). 11752141 10.1128/JVI.76.2.507-516.2002 PMC136834

[b39] Liu D. *et al.* Origin and diversity of novel avian influenza A H7N9 viruses causing human infection: phylogenetic, structural, and coalescent analyses. The Lancet 381, 1926–1932 (2013). 10.1016/S0140-6736(13)60938-1 23643111

[b40] McCullers J. A., Hoffmann E., Huber V. C. & Nickerson A. D. A single amino acid change in the C-terminal domain of the matrix protein M1 of influenza B virus confers mouse adaptation and virulence. Virology 336, 318–326 (2005). 15892972 10.1016/j.virol.2005.03.028 PMC2737340

[b41] Jackson D., Hossain M. J., Hickman D., Perez D. R. & Lamb R. A. A new influenza virus virulence determinant: the NS1 protein four C-terminal residues modulate pathogenicity. Proceedings of the National Academy of Sciences 105, 4381–4386 (2008). 10.1073/pnas.0800482105 PMC2393797 18334632

[b42] Ehrhardt C., Wolff T. & Ludwig S. Activation of phosphatidylinositol 3-kinase signaling by the nonstructural NS1 protein is not conserved among type A and B influenza viruses. Journal of virology 81, 12097–12100 (2007). 17715214 10.1128/JVI.01216-07 PMC2168765

[b43] Guan R. *et al.* Structural basis for the sequence-specific recognition of human ISG15 by the NS1 protein of influenza B virus. Proceedings of the National Academy of Sciences 108, 13468–13473 (2011). 10.1073/pnas.1107032108 PMC3158222 21808041

[b44] Sridharan H., Zhao C. & Krug R. M. Species Specificity of the NS1 Protein of Influenza B Virus NS1 Binds only human and non-human primate ubiquitin-like isg15 proteins. Journal of Biological Chemistry 285, 7852–7856 (2010). 20093371 10.1074/jbc.C109.095703 PMC2832935

[b45] Wright P. F. *et al.* The interferon antagonist NS2 protein of respiratory syncytial virus is an important virulence determinant for humans. Journal of Infectious Diseases 193, 573–581 (2006). 16425137 10.1086/499600

[b46] Brydon E. W., Morris S. J. & Sweet C. Role of apoptosis and cytokines in influenza virus morbidity. FEMS microbiology reviews 29, 837–850 (2005). 16102605 10.1016/j.femsre.2004.12.003

[b47] Van Reeth K. Cytokines in the pathogenesis of influenza. Veterinary microbiology 74, 109–116 (2000). 10799783 10.1016/s0378-1135(00)00171-1

[b48] Conn C. *et al.* Cytokines and the acute phase response to influenza virus in mice. American Journal of Physiology-Regulatory, Integrative and Comparative Physiology 268, R78–R84 (1995). 10.1152/ajpregu.1995.268.1.R78 7530928

[b49] Moran T. M., Isobe H., Fernandez-Sesma A. & Schulman J. L. Interleukin-4 causes delayed virus clearance in influenza virus-infected mice. Journal of virology 70, 5230–5235 (1996). 8764032 10.1128/jvi.70.8.5230-5235.1996 PMC190479

[b50] Baumgarth N., Brown L., Jackson D. & Kelso A. Novel features of the respiratory tract T-cell response to influenza virus infection: lung T cells increase expression of gamma interferon mRNA *in vivo* and maintain high levels of mRNA expression for interleukin-5 (IL-5) and IL-10. Journal of virology 68, 7575–7581 (1994). 7933145 10.1128/jvi.68.11.7575-7581.1994 PMC237205

[b51] Monteiro J. M., Harvey C. & Trinchieri G. Role of interleukin-12 in primary influenza virus infection. Journal of virology 72, 4825–4831 (1998). 9573248 10.1128/jvi.72.6.4825-4831.1998 PMC110027

[b52] Hamelin M.-È. *et al.* Oseltamivir-resistant pandemic A/H1N1 virus is as virulent as its wild-type counterpart in mice and ferrets. PLoS pathogens 6, e1001015 (2010). 20661429 10.1371/journal.ppat.1001015 PMC2908621

[b53] Huang S. S. *et al.* Pathogenic influenza B virus in the ferret model establishes lower respiratory tract infection. Journal of General Virology 95, 2127–2139 (2014). 24989173 10.1099/vir.0.064352-0 PMC4165929

[b54] Mänz B., Schwemmle M. & Brunotte L. Adaptation of avian influenza A virus polymerase in mammals to overcome the host species barrier. J Virol 87, 7200–7209 (2013). 23616660 10.1128/JVI.00980-13 PMC3700283

[b55] Naffakh N., Tomoiu A., Rameix-Welti M.-A. & van der Werf S. Host restriction of avian influenza viruses at the level of the ribonucleoproteins. Annu. Rev. Microbiol. 62, 403–424 (2008). 18785841 10.1146/annurev.micro.62.081307.162746

[b56] Li Z. *et al.* Molecular basis of replication of duck H5N1 influenza viruses in a mammalian mouse model. J Virol 79, 12058–12064 (2005). 16140781 10.1128/JVI.79.18.12058-12064.2005 PMC1212590

[b57] Paterson D., te Velthuis A. J., Vreede F. T. & Fodor E. Host restriction of influenza virus polymerase activity by PB2 627E is diminished on short viral templates in a nucleoprotein-independent manner. J Virol 88, 339–344 (2014). 24155385 10.1128/JVI.02022-13 PMC3911742

[b58] Gabriel G. & Fodor E. Molecular Determinants of Pathogenicity in the Polymerase Complex. Influenza Pathogenesis and Control-Volume I, 35–60 (2014). 10.1007/82_2014_386 25033751

[b59] Fan S. *et al.* Amino Acid Changes in the Influenza A Virus PA Protein That Attenuate Avian H5N1 Viruses in Mammals. Journal of virology 88, 13737–13746 (2014). 25231317 10.1128/JVI.01081-14 PMC4248959

[b60] Sutton T. C. *et al.* Airborne Transmission of Highly Pathogenic H7N1 Influenza Virus in Ferrets. Journal of virology 88, 6623–6635 (2014) 24696487 10.1128/JVI.02765-13 PMC4054360

